# Metronidazole-Induced Neurotoxicity Developed in Liver Cirrhosis

**DOI:** 10.4021/jocmr893w

**Published:** 2012-07-20

**Authors:** Takatsugu Yamamoto, Koichiro Abe, Hajime Anjiki, Taro Ishii, Yasushi Kuyama

**Affiliations:** a Department of Internal Medicine, Teikyo University School of Medicine, 2-11-1 Kaga, Itabashi-ku, 173-8605, Tokyo, Japan

**Keywords:** Metronidazole, Encephalopathy, Liver cirrhosis

## Abstract

A 68 year-old-male with hepatitis C-positive liver cirrhosis was admitted because of liver abscess. After metronidazole was initiated against the infection, mental disturbance appeared. Hepatic encephalopathy was suspected at first, however, the brain MRI showed hyperintense lesion of the bilateral basal dendric nuclei which indicated metronidazole-associated encephalopathy. The symptoms became well after cessation of the drug. Metronidazole is a widely used medicine against various infections. Recent case reports describe that this medicine can induce reversible encephalopathy. However, there have been few reports regarding metronidazole-induced encephalopathy occurred in patients with cirrhosis. Here we report on a case of hepatic cirrhosis and abscess in which reversible metronidazole-induced encephalopathy developed.

## Introduction

Metronidazole (MNZ) is a widely used medicine against various infections. Recent case reports show that this medicine can induce reversible encephalopathy [1-4]. However, there have been few reports regarding MNZ-induced encephalopathy occurred in liver cirrhosis. Here we report on a patient suffered from liver cirrhosis with recurrent hepatic encephalopathy who developed MNZ-induced neurotoxicity.

## Case Report

A 68 year-old-male, who had suffered from liver cirrhosis due to hepatic C virus infection, was referred to Teikyo University hospital (Tokyo, Japan) in August 2004, because of continuous fever rising. He had several hospitalization episodes for the treatment of hepatocellular carcinoma including transarterial chemo-embolization therapy, and recurrent hepatic encephalopathy in recent five years. On admission, the body temperature reached up to 39.7 °C, the blood pressure was 105/72 mmHg, the heart rate was 95 beats/minutes, and inflammatory change and marked hepatic dysfunction was seen in the blood tests (Table 1).

**Table 1:** Laboratory Data

Blood cell	Counts
RBC	402 x 10^4^/µL
Hb	13.3 g/dL
Ht	39.2%
Plt	2.3 x 10^4^/µL
WBC	13, 000 /µL
neutrophil	90%
PT	30.0%
APTT	67.8 s

**Chemistry**	**Number**

TP	5.7 g/dL
Alb	2.6 g/dL
T-Bil	15.0 mg/dL
D-Bil	9.9 mg/dL
AST	461 IU
ALT	194 IU
LDH	323 IU
ALP	295 IU
γ - GTP	285 IU
AMY	107 IU
BUN	50.9 mg/dL
Cr	1.4 mg/dL
Na	137 mEq/L
K	3.2 mEq/L
Cl	102 mEq/L
CRP	12.64 mg/dL
NH3	74 mg/dL
AFP	423 ng/mL
PIVKA-II	14 mAu/mL

**Figure 1:**
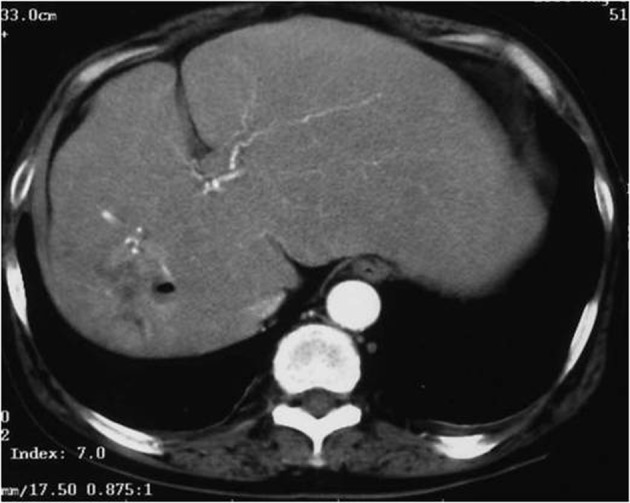
Computed tomography with contrast medium (arterial phase).

**Figure 2:**
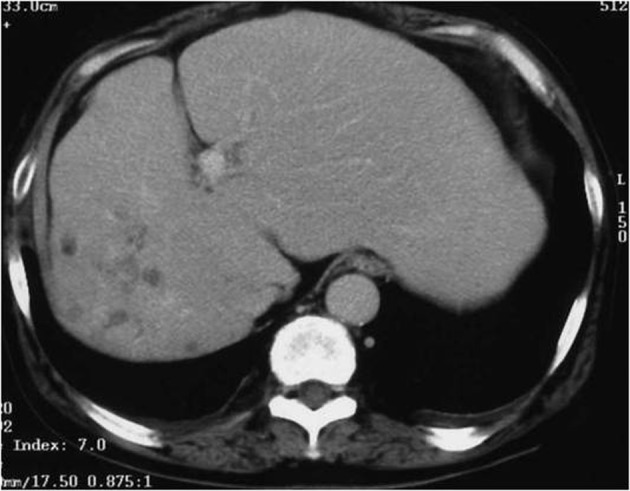
Computed tomography with contrast medium (portal phase). The presence of abscess was suspected at the right lobe of the liver.

**Figure 3:**
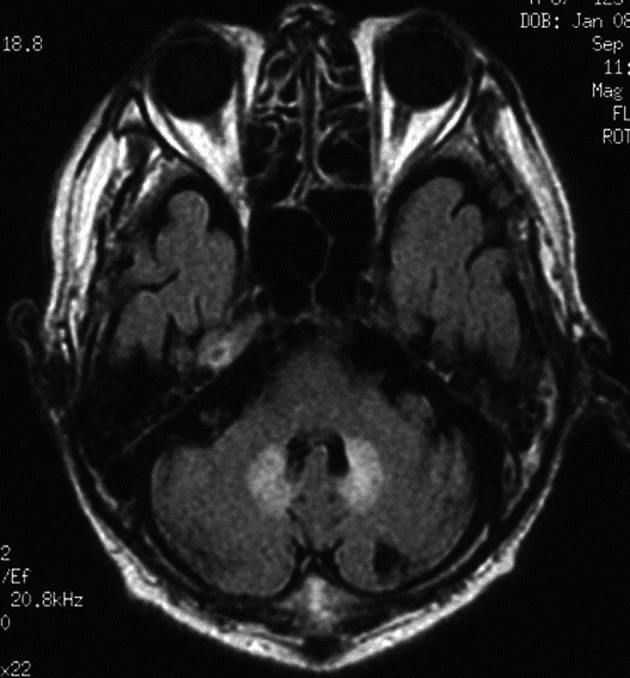
Brain MR imaging (FLAIR) showed hyperintense area of the bilateral basal dendric nuclei which was specific for those of metronidazole-associated encephalopathy.

**Figure 4:**
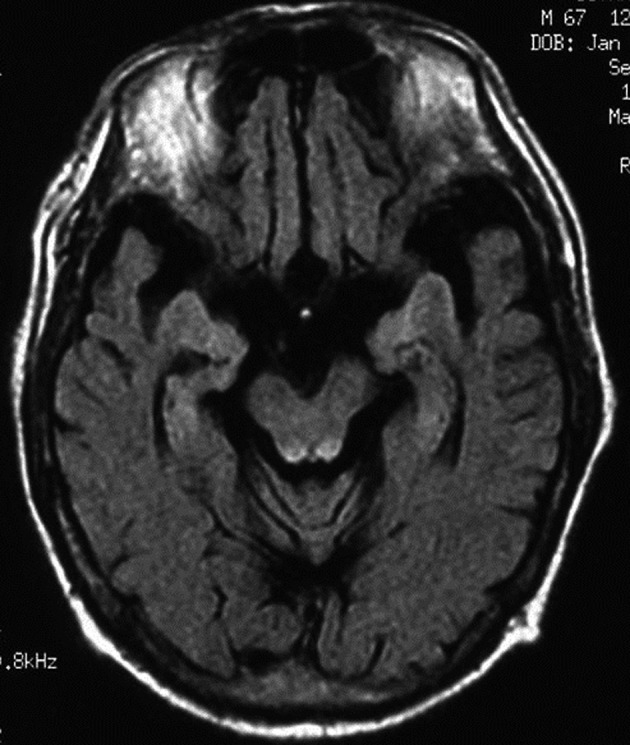
MR imaging (FLAIR) also showed hyperintense lesion of the brain stem.

**Figure 5:**
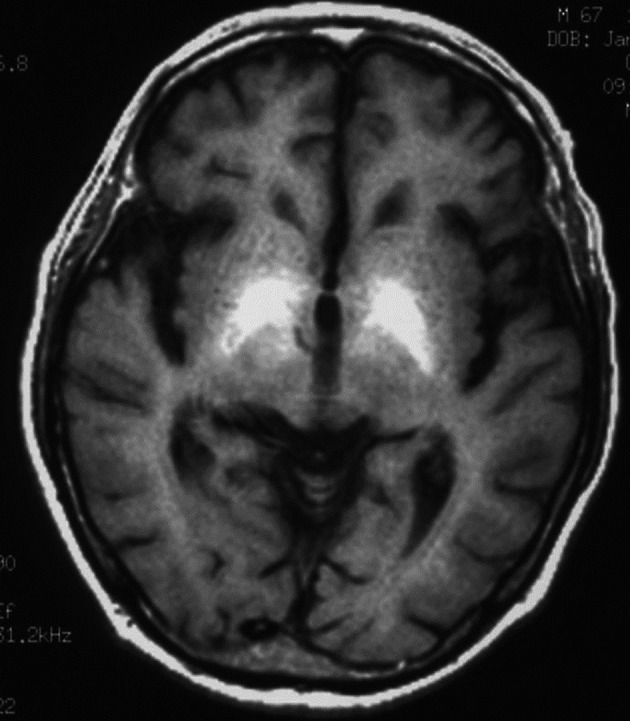
Brain MR imaging (T1WI) showed hyperintensity of basal nuclei indicating chronic hepatic encephalopathy.

**Figure 6:**
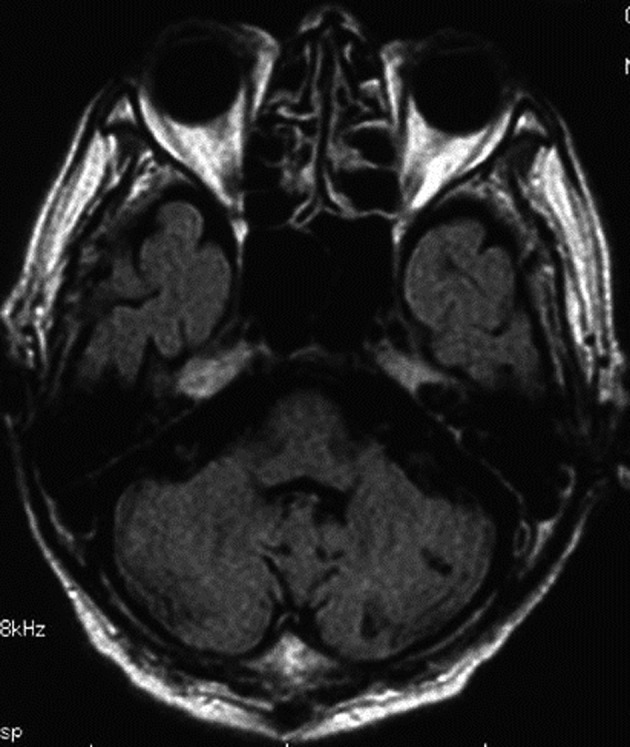
The hyperintensity of the dendric nuclei in MRI was disappeared one month later.

Abdominal computed tomography with contrast medium suggested the presence of liver abscess at the right lobe of the liver (Fig. 1, Fig. 2 ). Blood culture showed *Klebsiella pneumonia* and *Clostridium perfringens*, then imipenem and clindamycin was administered at first. Since the inflammatory change continued, clindamycin was changed to MNZ 1500 mg/day. Infection began to be improved after initiation of MNZ, however, speech and gait difficulty with mental disturbance suddenly appeared on the 19th day after MNZ administration. Although hepatic encephalopathy was suspected at first, the serum ammonia level did not show higher value than ever (56 mg/dL) and neurological findings were not compatible with the disease. Brain MRI showed hyperintense lesion of the bilateral basal dendric nuclei which was specific for those of metronidazole-associated encephalopathy (Fig. 3, Fig. 4), as well as hyperintensity of basal nuclei showing chronic hepatic encephalopathy (Fig. 5). After MNZ was ceased, the neurological abnormalities were improved gradually. MR image showed disappearance of hyperintensity of the dendric nuclei one month later (Fig. 6). The patient was discharged by walking alone three months later.

## Discussion

Metronidazole is a widely used antimicrobial agent against several infections due to *Clostridium difficile*, amoeba, *Helicobacter pylori*, and so on. The serious adverse effects of the drug include bone marrow suppression, liver or renal injury, and peripheral nerve injury. Incidence of the side effect regarding central nerve system is considerably rare, however, recent case reports showed reversible neurological disorders diagnosed by specific MRI of the finding of hyperintensity at the dendric nuclei and the brain stem [1-4]. Although the pathophysiology of the phenomenon remains unclear, some have suggested the RNA binding, DNA binding of intermediate metabolites of MNZ, or the modulation of gamma-aminobutyric acid receptor as possible mechanisms [5-7]. In the present case, severe liver dysfunction due to cirrhosis would facilitate the occurrence of the side effects because MNZ is mainly metabolized by the liver [8, 9].

Diagnosis of the disease can be made by the specific MR imaging mentioned above. Regarding this patient, although past history of hepatic encephalopathy made us think the recurrence at first, the neurological findings were different from the condition. Effective treatment for MNZ-induced neurotoxicity is cessation of the drug. Although severely ill case was reported, it recovered finally. The recovery of this case seemed slower than other reported cases, probably because the liver dysfunction prolonged metabolism and excretion of the drug.

In conclusion, we experienced MNZ-induced encephalopathy developed in patients with liver cirrhosis. Brain MRI demonstrated the specific finding of the hyperintensity at the dendric nuclei, which seemed good tool for its diagnosis and evaluation of the improvement. Since patients with liver dysfunction are feasible for the adverse event of MNZ, physicians should pay attention to this possible neurological side effect.
